# The regulation of acupuncture in France and the UK: Shifts and fragmentation in contrasting healthcare systems

**DOI:** 10.1177/0968533220903373

**Published:** 2020-03-13

**Authors:** Emilie Cloatre, Francesco Salvini Ramas

**Affiliations:** University of Kent, UK

**Keywords:** Acupuncture, public health, regulation, standardisation, complementary and alternative medicines

## Abstract

This article explores the regulation of acupuncture in the UK and France. It focuses on the dilemmas such regulation has raised, and the effects of two contrasting approaches to the regulatory organisation of acupuncture within healthcare systems on practices and care. Although the question of how acupuncture, like other complementary, alternative or traditional therapies, should be regulated has often been reduced to a question of scientific knowledge, it is also dependent on the intricacies of national health system governance, state rationales and professional identities. France and the UK provide exemplary instances of contrasting systems, in which each of these factors has come to shape the regulation of the highly heterodox practice that is acupuncture. Overall, exploring the challenges of regulating acupuncture provides useful perspectives on how the make-up of legitimate therapies is constituted in particular healthcare contexts.

## Introduction

In this article, we explore the regulation of acupuncture in the UK and France. We focus on the challenges such regulation has raised, and the effects that two very contrasting approaches to the regulatory organisation of acupuncture within healthcare systems have had on practices and care. Although the question of how acupuncture, like other complementary, alternative or traditional therapies, should be regulated has often been reduced to a question of scientific knowledge, it is also dependent on the intricacies of national health system governance, state rationales and professional identities. France and the UK provide exemplary instances of contrasting systems, in which each of these factors has come to shape the regulation of the highly heterodox practice that is acupuncture. On the one hand, the UK has opted for a bureaucratic and decentralised system of regulation, where a key concern and dividing line is the issue of public funding. There, professional regulation operates as a separate matter from that of scientific validity and complicates the question of who should be entitled to provide health and care services. France, on the other hand, relies on a system in which professions and legitimacy are deeply entangled: the core question that has so far underpinned the organisation of acupuncture is that of the monopoly that doctors and health professionals are, by law, understood to exercise over bodily health. The issue of public funding, though also very relevant, can be seen to operate as a secondary (yet interrelated) level of debate to that of internal and professional struggles. In both cases, however, regulation creates a disparate landscape, that complicates the heterodox nature of acupuncture as practice, but also comes to reinforce and illustrate core differences between national systems of care. France remains attached to a particular understanding of medical professions as guarantors of bodily well-being that is less marked in the UK, where legitimacy is essentially about institutions and their management.

This translates into contrasting landscapes for the practice of acupuncture in France and in the UK, which matter to patients’ care. A patient seeking acupuncture in the UK will have two main routes offered to them. They might be able, for a limited range of conditions (including migraines, chronic pain and joint pain), and depending on local circumstances, to have some sessions prescribed to them by their doctor. If this happens, they will usually be referred to a physiotherapist who will offer a limited number of sessions, in an environment and through methods and languages that are likely to feel very clinical. Patients who are not able to benefit from the National Health Service (NHS) offering, or indeed are seeking a different kind of acupuncture – with a more ‘traditional feel’ maybe – will also be able to approach private practitioners. Those are likely to be trained as professional acupuncturists (rather than, for example, physiotherapists with a training in acupuncture), and many (though not all) will be part of a voluntary professional association. In France, the same patient will see two main routes open to them. First, they may choose to visit a doctor-acupuncturist: if so, their consultation (or at least part of it) will be refunded through the social security system. Indeed, in most cases, consulting such doctors may be the only legal route to acupuncture (with the exception of pregnancy and labour, for which midwives with relevant qualifications can intervene). But patients will also be able to find practitioners trained in acupuncture who are neither doctors nor midwives, and indeed are not practicing legally. The latter, however, is not necessarily obvious to patients, since they will find these practitioners through simple Internet or directory searches as they would with any other professionals, including doctors. In France, the experience may also be less easily divided between the clinical and the ‘traditional’, as both doctors and other practitioners divide themselves against these categories in quite disparate ways. Behind each of these apparently mundane variations, divisions and pathways reflect much broader professional and institutional contrasts between the practice of medicine, and of its borders, in France and in the UK. Acupuncture also gives us specific insights into how the question of expert knowledge navigates in and out of those institutions and fields of practice. At the same time, and in spite of their differences, the two case studies illustrate that acupuncture continues to unsettle regulatory systems: it remains a site of friction that each system manages differently and keeps rewriting over time.

This article is divided in three parts: we start by providing contextual and conceptual background to our analysis, reviewing relevant sociological and historical literature, before turning to how we approach regulation in this article, and, briefly, the methods on which this research is based. We then present the empirical context of acupuncture regulation in France and the UK, focusing on the contemporary but positioning it against the context of its evolution, in particular since the 1990s. Finally, we discuss what looking at UK and French acupuncture side-by-side can suggest of the process of regulating the place of practices that ‘do not quite fit’ within health systems.

## The context of acupuncture in France and the UK

The place of acupuncture in health practices in Europe has long been a subject of both scholarly and policy interest. Earlier research has shed some light onto the heterogeneous nature of acupuncture, its negotiated place in public understandings and health systems in Europe, and the challenges it continues to raise in terms of tensions around the nature of ‘valid’ knowledge in healthcare. In this section, we start by briefly reviewing this literature, before sketching some of the broader questions we seek to interrogate by looking at the regulation of acupuncture in France and in the UK.

### Acupuncture: Histories and sociology

Acupuncture has been a relatively common practice in Europe since the 1970s and is now considered to be one of the most ‘settled’ forms of alternative/complementary medical practice. Its history on the continent however goes back much further, with its import intriguing and unsettling medical establishments throughout the 19th and early 20th century.^1^ Early interactions illustrate vividly the tensions between ways of knowing that would remain at the core of contemporary dilemmas: while some doctors and practitioners saw acupuncture as a new way to approach healing, philosophically as well as therapeutically, others were prompted to seek to translate the practice of acupuncture into known medical paradigms.^2^ To this day, tensions between a ‘traditionalist’ current in acupuncture, that considers acupuncture as non-reducible to scientific logic, and a ‘scientist’ current, that sees its translation into science as essential to its integration into medical practices, continue to coexist both within and across professional associations.^3^ The position of acupuncture in healthcare navigates across these identities. As we return to below, acupuncture is sometimes presented as a scientific practice, which can be isolated from its traditional roots and adapted to the methods of the biomedical system. Here acupuncture is legitimate if and when it is proven and practiced in terms familiar to science.^4^ Yet acupuncture’s legitimacy is also negotiated through its difference with science, and because of its philosophical roots as well as its oriental origins that continue to appeal to patients.^5^ This is not specific to Europe, and authors have pointed to such tensions in other contexts, from China and Korea,^6^ to Europe and North America.^7^

Such dual identity has two sets of implications that permeate the remainder of our analysis. First, acupuncture is a heterogeneous practice. Sociologists have shown, for example, how for both practitioners and patients acupuncture can rest on a multiplicity of ontologies and of therapeutic expectations.^8^ Learning from acupuncture as an epistemological system, Lin suggests we have to read the organisation of acupuncture as part of a multiple world, not focusing on the rationales themselves, as separated wills, but on their intertwining.^9^ As we will see, in the context of France and the UK, such multiplicity and intertwining cut across sites, practices and practitioners. Second, tensions arise as to questions of regulation and legitimacy: while healthcare systems tend to rely on scientific processes of evidence-making to negotiate the place of a particular treatment, the dual nature of acupuncture makes it more difficult to decide how legitimacy should be settled and by whom. Authors have approached the organisation of acupuncture as a therapeutic practice in specific institutional systems in terms of *transmission*,^10^ approaching knowledge patterns around acupuncture as hybrid, as well as unsettling. Acupuncture, as a field of knowledge, can thus be approached as never quite fitting, yet challenging the contemporary health complex and its regulation, dominated by biomedical practices and rationales.

Fundamentally, at stake in debates around the place of acupuncture, and its regulation, is the question of knowledge and how to discipline or enable it. Rather than a practice or knowledge system, acupuncture may best be defined as a ‘set of heterogeneous bodies of knowledge that differ between societies, organisations and courses, each promoting a different kind of acupuncture knowledge’.^11^ As a result, the modernisation of acupuncture cannot be analysed only as a process of being ‘made scientific’, but rather as an uncertain normalisation of alterity that proceeds by translating the epistemological world of acupuncture out of its own cultural, spiritual and even material complexity. In Wahlberg’s analysis, this normalisation proceeds by recoding the practice, in terms of regulation and medical rationale, engaging with a different social and cultural context, in Western societies, and disciplining it, ‘as a means of enhancing their capacities, extorting their forces and securing certain distributions of them’.^12^

### Acupuncture in France and the UK: Interrogating regulation, law and institutions

Although acupuncture has been explored extensively as social and medical practice, its regulation has been given less scholarly and critical attention. Yet regulation is central to the mechanisms at play when acupuncture is brought into biomedical-based health systems. For example, the process of transmission is characterised as a move from exclusion or alternative, into integration and normalisation. It may include attempts to translate, including through sociological language, the logic of the meridian system of intervention at the basis of acupuncture to the biomedical debate.^13^ Such mediation is dependent on the pragmatic organisation of education, training and lobbying through regulation that others have explored in depth.^14^ But these movements towards integration (however conditional) are also accompanied by a process of differential inclusion, where acupuncture may carve itself a space within the main healthcare system, but always through a certain negotiation.^15^ There, the epistemological definition of its validity, and its ability to enter new spaces, is mediated significantly by the legal organisation of professions, institutions and materials.^16^ Scholarship so far has paid less attention to how contrasting models of law and regulation for acupuncture impact such movements and the practice of acupuncture, or indeed what they can tell us about states rationales embedded in regulatory systems.

Approaching law and regulation as part of therapeutic practices, and the delivery of care, requires particular analytical tools. In this article, we rely primarily on Science and Technology Studies (STS)-inflected approaches to law and regulation.^17^ Rather than imagining law as a stand-alone system that responds to and normatively shapes medicine and science, we approach law and regulation as one set of social and institutional rules that co-produce medical practices.^18^ Furthermore, law and regulations embed state identities, professional traditions and lived relationships of power: health systems are each conditioned with and through law by broader state histories and identities.^19^ Conversely, regulatory systems and their everyday functioning can reveal aspects of those identities and traces of historical remnants and influences.^20^ Finally, the relation between law and knowledge can be read as a process of contingent co-production: although much regulatory debates in the field are approached as issues to be settled through scientific knowledge, regulation cannot be approached as simply resting on a rational embracing of such knowledge. Indeed, institutional organisations, the historical shaping of relations between state and professions and the distribution of roles across disparate fields of practice, all shape approaches to acupuncture as heavily as scientific settlement might. At the same time, as the regulation of acupuncture in different contexts is explored, it becomes clear that, far from being a discreet matter of knowledge to be settled only through evidence, acupuncture is a social and organisational conundrum that needs to be apprehended as a heterogeneous practice of care. This heterogeneity opens the door for contingent and multidirectional relationships to regulations: overall, regulation finds itself at the crossroad of inscribed histories and systemic shaping, and of the minute contingencies, responses, adjustment and resistance that are generated through everyday practice and opportunities.

## Methods

The research on which this article is based was drawn from documentary analysis and semi-structured interviews, conducted between March 2017 and November 2019.^21^ The data on acupuncture presented here form part of a larger project on the regulation of traditional and alternative medicines, in the context of which we conducted a wider set of interviews in France and the UK (over 60 to date, but our research is ongoing).^22^ Our argument here is based primarily on a specific set of interviews more specifically relevant to acupuncture. Because of our focus on regulation and institutions, in the project as a whole and as far as acupuncture is concerned, we focused primarily on interviewing actors that had a specific role within the institutional framework of acupuncture in France and the UK, as well as some practitioners who had also previously engaged with the regulatory systems we scrutinised. In the UK, those most relevant to this article included interviews with institutional representatives and spokespersons of the Professional Standards Authority (PSA) for Health and Social Care and Complementary and Natural Healthcare Council (CHNC), British Medical Acupuncture Society and British Acupuncture Council (BAcC), and five regulated and non-regulated acupuncture practitioners. In France, relevant interviews were with representatives of four (medical and non-medical) acupuncturists associations, six practicing acupuncturists (both doctors and non-doctors), the director of one specialist medical unit, a civil servant who had been involved in previous regulatory debates, as well as representatives of institutions framing the broader debate (including one member of the Ministry of Health and two representatives of the Mission Interministerielle de Vigilance et de Lutte Contre les Dérives sectaires (Miviludes)). The documents we analysed alongside these interviews comprised both state regulatory documents, and the numerous codes of conduct, reports and statements produced by associations of practitioners, as well as reports and white papers, both in the UK and France. Relevant case law and media reports were also analysed to provide broader contextualisation. Although we have in mind the longer historical background of acupuncture, our analysis is primarily based in the study of contemporary regulation, mostly since the early 2000s. These data were read in conjunction with the broader sociological literature that has interrogated more closely the practices and everyday experiences of patients and practitioners. Finally, although not used directly as primary data in this article, the broader set of interviews carried out in this project have contributed to shaping the questions and approaches embedded in our analysis of acupuncture.

In the sections that follow, we provide an overview of the UK and French regulatory systems for acupuncture and map them against practices and delivery of care. We then turn to analysing what those two models can suggest of the type of frictions and dilemmas acupuncture continues to raise for regulators, and of the broader interface between state, institutions, and therapeutic regulation in France and the UK. For clarity, we present them in turn before turning to analysing side-by-side some of their notable features.

## Negotiating the place of acupuncture in the UK

We describe the regulation of acupuncture in the UK as ‘fragmented’. Possibilities of practicing acupuncture are not regulated strictly through law and are organised principally through two different circuits: one centred around the provision of public healthcare and the other private and marked-based. The edges of each circuit however, overlap, making the differentiations between the two less visible. In their practice, each actor intersects differently with a number of institutional bodies and regulations, complicating the UK system. In the sections that follow, we explore some of these movements and the decentralised and multiple system that regulates contemporary UK acupuncture. Before turning to these recent movements, however, it is worth noting briefly how those have shifted from the more ‘experimental’ phase of previous decades.

### The rise of contemporary acupuncture

While the regulation of acupuncture itself has been relatively stable (if hotly contested) in France since the popularisation of acupuncture in the 1970s, the regulatory system in the UK as it exists today has been shaped by several shifts and key moments. Since the 1970s, acupuncture has been used by a broad public in the UK, in physical and mental well-being. In the context of the geopolitics of medicine and science after the Second World War, acupuncture occupied a place of interest both as a result of the long tradition of (post)colonial knowledge transfer and as a result of the new flows of counter-culture, and, in some measure, Orientalism.^23^ Through the 1980s, the presence of acupuncture in the UK, in everyday practices and in the public imaginary, continued to establish itself in several sites and networks of collaboration and learning, although often in communities and groups on the fringe of mainstream healthcare. For example, until the late 1990s, acupuncture was particularly used in the contexts of mental healthcare, HIV and hepatitis care, as well as drug addiction therapy.^24^ Progressively, it then branched out more explicitly into the public institutional frame, leading to the opening of experimental services in different segments of the ‘state’, such as NHS clinics and local council centres for drug addiction, or rehabilitation homes.^25^

Noticeably, and, as we will return to, in significant contrast with events in France, many of these early experiments were not led by doctors, but by other ‘ancillary’ figures such as physiotherapists, nurses and other health professionals, together with other non-biomedical actors. As many of our interviewees account, regulation in this period was still latent and the management of practices decentralised and tentative: experimental practices operated both within and outside the NHS; actors would navigate these systems through localised agreements. A striking feature is that these sites straddled institutional and professional boundaries between acupuncture and biomedical practice, facilitating the emergence of new practices, most notably in situations where Western medical knowledge was less stable or less successful.

The early 2000s represented a moment in which the attempt to regulate Complementary and Alternative Medicine (CAM), and acupuncture within them, became an explicit policy goal, as a result of the National Health Service Reform and Health Care Professions Act 2002, and of a broader regulatory effort to engage with CAM since a 2000 House of Lords report (which had considered acupuncture’s statutory regulation a priority).^26^ Two different circuits of regulation emerged from such efforts. The first was a result of the managerial organisation of care in the NHS, and the second was part of the rising system of regulation of alternative therapies through the Council for Healthcare Regulatory Excellence (CHRE) and a market logic.

First, in 2008, following the ‘Department of Health Steering Group on the Statutory Regulation of Practitioners of Acupuncture, Herbal Medicine, Traditional Chinese Medicine and Other Traditional Medicine Systems Practised in the UK’,^27^ proposals emerged which sought to regulate the relationship between the NHS and the practice of acupuncture through the Health and Care Professions Council (HCPC), a body created in 2002 and mandated with regulating and monitoring therapeutic professions ranging from social work, to art and occupation therapists and, relevant to our case, physiotherapists. The proposal of the DH Steering Group was to frame acupuncture through a statutory system, identifying physiotherapy as the profession through which to articulate governance in acupuncture, by ‘setting standards, protecting commonly recognised professional titles and providing a way in which complaints can be dealt with fairly and appropriately’.^28^

Second, before and after the attempt to place acupuncture within the ambit of the HCPC and alongside this regulatory path for acupuncture ‘by health professionals’, other paths were opened for those who were not health professionals and who tended to define themselves as ‘traditional acupuncturists’. This was notably through the CHRE (later known as the Professional Standards Authority (PSA)), set up as a meta-regulator, to oversee professional associations including those of osteopathy, midwifery, cosmetic, pharmacy or dental care, as well as acupuncture, against specific standards: the quality of services and facilities, as well as the internal regulation of the registry. In other words, the CHRE (and PSA) assessment had no relation to the body of knowledge embedded in acupuncture and focused instead on the regulation of professional behaviour: it did not get involved with questions of validity of knowledge and did not intervene in the circuits of purchase and provision of services of the NHS.^29^

In practice, however, these paths collided: different governments through the 2000s supported one or another way of regulating acupuncture practice, tangling up norms, protocols and actors.^30^ This led to the failure both of the DH Steering group proposal of 2008, to regulate acupuncture only through the physiotherapist profession and within the NHS, and the CHRE’s attempt of creating a statutory professional registry for acupuncture, as a service available through the private market.

### The multiple regulation of acupuncture

The overlapping of these two regulatory bodies, as well as disagreements as to which should be the reference to regulate acupuncture, led to the creation of a highly fragmented model, endorsed by the 2011 Secretary of State for Health policy paper titled ‘Enabling Excellence’. This article recommended the creation of voluntary, rather than statutory registers, as they could offer a ‘more proportionate way of balancing the desire to drive up the quality of the workforce…’,^31^ and a focus on the guidance of the National Institute for Health and Care Excellence (NICE) to regulate acupuncture in the NHS. Here we analyse the functioning of this dual system in more detail.

#### Regulating the place of acupuncture in the NHS

A first framework of regulation has effectively aimed to integrate (some) acupuncture within the NHS. This is the regulation of the ‘biomedically recognised’ use of acupuncture which, since 2008, is evaluated by NICE, implemented by the NHS, and operated principally through the HCPC, and the Acupuncture Association of Chartered Physiotherapists. NICE, a Non-Departmental Public Body responsible for providing evidence-based guidance on health and social care, has been increasingly monitoring and changing its guidance on the use of acupuncture since 2008, when three reports were published, until today, when approximately 10 reports are published annually, addressing the use of acupuncture in relation to more than 30 conditions.^32^ Perhaps unsurprisingly, given the breadth and focus of NICE’s activities, the presence of acupuncture is marginal in NICE documents. Acupuncture is mentioned principally as a non-supported practice for a number of conditions in which other biomedical treatments are provided and funded, such as mental health conditions. Additionally, NICE guidance principally permits acupuncture for some musculoskeletal treatments but advises explicitly against the publicly funded provision of acupuncture for other specific treatments, such as post-partum management or low back pain.^33^

On the basis of this guidance and its translation to the NHS through Clinic Knowledge Summaries (CKS), doctors and other NHS health professionals can only prescribe acupuncture for certain specific conditions. NICE regulated practices are ‘referable’ by NHS professionals, and they are provided mostly through external providers within NHS Community Clinics, regulated by the HCPC, where different health professionals operate. In these sites, acupuncture – especially for musculoskeletal treatments – is delivered by physiotherapists listed in the Acupuncture Association of Chartered Physiotherapists (AACP) who are normally trained in Western acupuncture. The AACP organises more than 5000 physiotherapist practitioners in NHS community clinics, financed by public resources through the referral system. AACP members cover thousands of patients and can also privately provide acupuncture for other purposes, and in the same clinics, albeit without public funding.

#### Regulating acupuncture as profession: Gaps and hybridities

Besides its regulation within the NHS, acupuncture is also regulated as an independent profession: those who are not practicing in the NHS (or by referral) are therefore regulated through a separate route of professional regulation. This can be either through voluntary registers affiliated to the PSA and/or through the more general set of rules that regulate commercial practices. Acupuncturists are free to practice privately without subscribing to registers, though within the limits imposed by common law on bodily and psychological harm. In these two framings, acupuncture is regulated not as a healing practice but as a commodity in the market, in order to make its circulation safer for the consumer. However, in both scenarios, the deployment of regulation is confused, and private and non-healthcare practices trespass the tiers of regulation. Here, because of our focus on regulatory strategies and their limitations, we will deal with the first scenario, leaving aside the ‘unregulated’, which raises a set of questions beyond the scope of this article.

Looking at the voluntary registers’ regulation, the practice of such protocols has produced a number of unexpected consequences, leading to the emergence of different spaces and paths for accessing acupuncture. Although regulation through the PSA is not supposed to be about therapeutic legitimacy (and its representatives are very clear on this), acupuncturists and other CAM practitioners have often blurred the boundary between professional and therapeutic legitimacy. The most prominent actor in this space is the BAcC, which unified five different former organisations in 1995 and became a voluntary registry under the PSA in 2013: this fed into its effort to present private acupuncture as a professionalised and legitimate agent in health and social care. It has more than 2000 members, which it accredits and monitors; it also monitors training schemes in a number of private institutions, in order to maintain quality standards in the continuous training that is mandatory for its members. BAcC regulates and organises non-biomedical practitioners, which provide their services privately in places increasingly separated by the NHS referral system. This impacts on possibilities of access to those who can afford it.^34^

Since the BAcC is meta-regulated by the PSA’s commercial and non-biomedical standards, its members are regulated as commercial professionals, but not as healthcare professionals. They can access the mechanisms of evaluation and monitoring that would allow them to access the circuits of NHS only if they are also members of the AACP, and therefore physiotherapists recognised and organised through HCPC regulation, or otherwise, have to follow an opaque and expensive path to be recognised as NHS non-HCPC professional providers.

At the same time, even if they are side-lined from the NHS system, the segmentation between NICE, CKS and NHS leaves a margin of manoeuvre to each of these agents. Where the NHS approach privileges science in accordance with the logic of evidence-based cost-efficiency principles in NICE guidance, the social and public function of NHS is also that of informing the public and this gives a broader space of recognition to acupuncture. In the NHS Choice web pages, for example, acupuncture is sponsored as a possible practice in a number of situations not recognised by NICE guidance, and the acupuncturists organised in the BAcC are publicly recognised by the NHS system as reliable actors in the practice of acupuncture: concretely, they are presented as the trustworthy actor for acupuncture practices, and acupuncture is defined as a relevant alternative therapy.

Overall, the governance of acupuncture is based not on the definition of strict borders but on the deployment of two separate yet, in practice, overlapping frameworks. On the one hand, the careful regulation of public resources for health through principles of proof and cost-effectiveness. On the other, the enhancing of accountability and transparency in self-regulating practices, as far as a broader ‘marketplace for consumers’ is concerned. As we return to in our discussion, this system of tiers and segments makes acupuncture a fragmented practice and leaves a number of gaps and uncertainties unresolved.

## France and the drawing of il/legal boundaries

France, much like the UK, has always had an ambivalent relationship to acupuncture. However, the expression of this ambivalence has been shaped both by pre-existing legal principles and by a particular relationship between state and the medical profession. Growing in popularity from the 1970s to the early 1990s, acupuncture was initially ring-fenced as something that could only be practiced by specialised doctors.^35^ Since the 1990s however, this monopoly (though still inscribed in law) has been increasingly challenged. At the same time, the question of if and how acupuncture was sufficiently scientifically proven to be subsidised by public funds, brought into hospitals, or more generally offered to patients, has continued to be debated.

The organisation and regulation of acupuncture in France has also always depended on the particular legal and political context of medicine, and notably the legal monopoly of medical doctors over bodily care.^36^ This has shaped the French regulatory system of acupuncture as one in which questions of legality, and of professional belonging, are central.^37^ At the same time, the everyday practice of acupuncture has deviated from what a strict reading of the law may suggest: acupuncture has escaped to new areas, where (more or less) discreet practices have destabilised the capacity of exclusion and control of the law. Where the law has appeared as less relevant to practice, however, questions of health financing have all but taken the more decisive regulatory role in shaping where and by whom acupuncture is provided as part of the French healthcare system.

### State law, medical monopoly and the place of acupuncture

The regulation of acupuncture in France is first and foremost dependent on the broader monopoly of doctors over ‘diagnosis’ and ‘treatment’ of bodily conditions. This is inscribed in the Code de la Santé Publique (Code of Public Health), which prohibits anyone other than a doctor, and, by means of exception for specific acts, other health professionals, from undertaking either of those activities.^38^ Anyone who is not medically qualified can in principle be found guilty of illegal medical practice, a crime that carries a potential penalty of up to 2 years imprisonment and a 30,000 euros fine (though case law suggests most cases result in much lower penalty). Exceptions have been built for other health professions, who may independently or under supervision seek to diagnose or treat, within the limits of their own professional codes and those of the law (including, since 2002, osteopaths). But acupuncturists have not emerged as such a recognised health profession, in spite of ongoing demands, which we return to below. Instead, the practice of acupuncture remains within the medical monopoly. The Ordre des Médecins (The College of Medical Practitioners) recognises it as a ‘medical orientation’ (since 1974). However, doctor-acupuncturists are not considered as ‘specialists’ (which affects, for example, the tariff on which the social security will contribute to their consultations).^39^ Midwives have successfully campaigned to be authorised to practice acupuncture, subject to adequate training, and indeed acupuncture has grown in popularity in obstetrics practice.^40^

Limitations to acupuncture practice, set as they are by the monopoly offered by law to medical doctors, are not specific to acupuncture, but impact any CAM treatment: it is the general principle that ‘only doctors can treat’ that is fundamental here. However, where other CAM practices can potentially straddle the boundary between health and well-being, arguing that what they provide is (legal) care/well-being/nutrition, rather than (illegal) health treatment, this is more difficult for acupuncturists. The act of piercing through the skin, when in a context of care, is considered as being inherently medical.^41^ The legal limitation on medical practice for non-doctors is based on a number of rationales – in addition to, undeniably, a particular strength of medicine as a profession within the French legal and political system. First, the law seeks to protect patients against the risk of inadequately trained practitioners intervening on their bodies: unsafe treatment, or treatment inadequately provided, could harm patients. Needles here are particularly ‘risky’ because of the dangers of breaking through the skin with inadequately sterilised instruments, or at the wrong site or depth. Second, the medical monopoly aims to protect patients against loss of opportunities: by limiting the domain of diagnosis and treatment to those who have particular qualifications, and are under particular institutional and professional oversight, the system aims to ensure that potential illnesses will not be ‘missed’, or medical treatment delayed, unnecessarily.^42^

Overall, based on the above principles, in theory at least, the landscape of French acupuncture should present a relatively clear border system in which acupuncture is available to patients through specialised sections of the medical community, and where others are prevented from offering what would effectively be the illegal use of the technique (and of medicine). In practice, however, the landscape of acupuncture has been much more ambivalent.

#### Acupuncture in France: Offerings and practices

Acupuncture in France can be practiced in private clinics and offices, or, more rarely but increasingly, in hospitals. The landscape is also made up of both doctor-acupuncturists and non-doctor/illegal therapists. Doctor-acupuncturists have for a long time had a relatively uncontested monopoly, and indeed, throughout the 1980s in particular, acupuncture has been considered a worthy route towards professional opportunities. Training, including in medical faculties grew exponentially from the mid-1980s, with the first Diplome Inter-Universitaire created in 1987.^43^ If this enabled acupuncture to secure its place within medical practice, such settlement was also limited and fragile: attempts to have acupuncture recognised as a full ‘spécialité’ by the Ordre des Médecins, which would have carried both prestige and financial implications for doctors, were never successful, and neither were efforts to increase the rate on which acupuncture sessions could be subsidised by public health insurance. These difficult negotiations also had an impact on the possibilities for acupuncture to be integrated into hospital care, where the mapping out of expenditures against medical acts is even more complex than in private practice. If acupuncture has to some extent ‘entered the hospital’, it remains relatively ad hoc, constrained to particular wards, notably for pain management and obstetrics (including through the impetus of midwifery’s access to acupuncture), and dependent on the interest of individual doctors or to some extent midwives.^44^ In addition to these hurdles, from the 1990s onwards, the monopoly of doctors, as we return to below, became contested by other non-medically trained acupuncturists. Finally, and most recently, doctor-acupuncturists, like others who provide forms of ‘alternative/complementary’ therapies, have been under increasing attack from within their profession. For example, in 2018, a campaign by a collective of doctors argued against the provision of any ‘unproven CAM’ by medical doctors and against their reimbursement. Given the regulatory restrictions on anyone other than a doctor providing CAM, the call seems effectively to be seeking for CAMs to be prohibited. The arguments of the campaign are based centrally on the lack of scientific evidence in support of CAM: CAM are presented as no more than quackery, misleading patients and representing a breach of professional duties by doctors. Although the primary target of the campaign seems to be homeopathy, acupuncture is dealt with a comparable level of suspicion and agglomerated in the overall concept of ‘fake medicines’ (with its own hashtag on social media) that the collective embraces.^45^ Here, the fact that CAM has been in France mainly within the monopoly of doctors seems to have made it even more vulnerable to such attacks from within the profession: the assumption that acupuncture should be reserved for doctors, inscribes it within a medical paradigm from which scientific evidence is hard to disentangle. At the same time, and as we return to below, such a campaign seems to leave aside some of the more complex levels of interface between scientific evidence and regulatory systems, or between what is proven and what should be ‘allowed’ (and by whom) or ‘removed’ from the public health system.

Alongside medical doctors practicing acupuncture, and despite clear boundaries drawn by state law, many non-doctors continue to practice ‘illegally’. This is not specific to acupuncture but widespread in CAM practices, with many therapists practicing on the verge of legality.^46^ While some choose to ‘negotiate’ with legality, seeking to present themselves as providing something other than ‘healthcare’, this is less possible for acupuncturists. This negotiation with il/legality is interesting at two levels. First, it creates a particular sphere of ‘formally unregulated practice’, that generates a degree of precarity both for practitioners (who may always be caught into accusations of illegal medical practice and prosecution) and for patients (who have little means of differentiating the professional standing of practitioners). Similarly, the fact that those practitioners operate ‘in the shadow’ of state regulation and of the healthcare system means that the sessions they provide cannot be subsidised by public health insurance, which impacts on both their income, and on access for patients. Similarly, this position ‘in the shadows’ of the healthcare system means that access to hospitals and public services for non-medical acupuncturists is nearly impossible. Second, this (albeit disparate) community of therapists has organised to try to erode in law as well as in practice the monopoly of doctors over the practice of acupuncture. Associations have been set up, and have become more established since the early 2000s, in order to provide both support for practitioners (e.g. legal advice, codes of practice, insurance schemes) and to create campaign groups to seek formal recognition by the state, and to be recognised as a profession able to practice alongside doctors.^47^

Overall, in the regulation of acupuncture practice in France since the 1990s, legitimacy has been negotiated at several levels – state law, professional regulations and everyday tactics of negotiation of il/legality. Against these negotiations, the question of scientific validity has inevitably emerged. Although it has represented a feature of both boundary making and state decisions since the 1970s, it has been particularly prominent in recent efforts to settle ongoing disputes.

#### Settling regulatory controversies through scientific evaluation

Contemporary conversations, from claims to counterclaims regarding acupuncture, have resulted in new efforts by the state to evaluate the ‘scientific value’ of acupuncture. The first step has been for the Haute Autorité de Santé to task the Institut National de la Santé et de la Recherche Médicale (National Institute of Healthcare and Medical Research, INSERM in its French acronym) with a systematic review of scientific evidence currently available, in order to assess to what extent the efficacy of acupuncture had been proven. It is expected that such evidence will then determine recommendations for its continuing financing, or its suspension. In 2014, the INSERM released a 212 page report with its conclusions.^48^ Overall, the authors of the report refrain from any certainty: instead, they point to discrepancies between what acupuncturists claim about the effects of acupuncture and what the scientific literature to date suggests, and call for new evidence and systematic testing. Yet the report also refrains from pointing conclusively to a complete lack of evidence, something upon which the FakeMeds collective have based their opposition to acupuncture. Overall, it seems to provide few new tools with which to settle the regulatory dilemmas around either the value of subsidising acupuncture, or indeed the more complex question of who should be able to practice it or how. In France, like in the UK, however, it is difficult to disentangle policy discourses from this appeal to science. In addition, the regulatory system as it stands, while enabling some degree of formal professional requirements and standards as far as doctors, midwives and hospitals are concerned, does not in practice have much influence on the many non-medical practitioners who offer acupuncture in spite of the law. Indeed, socio-legal scholarship may suggest that such diffuse practice is unlikely to lend itself to any efforts to prohibit. At best, tightening sanctions against practitioners may bring such offerings into more discreet spaces, but may consequently offer even less protection to patients. More thorough questions around regulation itself, including the approach that may be taken to non-biomedical acupuncturists and their place within the healthcare system as a whole, are unlikely to be settled through science. We return below to this question, analysing in parallel the role of science, knowledges, and professions within the regulation of practice.

## Analysis

Observing the regulation of acupuncture in France and the UK illustrates some obvious differences in state strategies, but also some comparable difficulties in ‘settling’ acupuncture within the broader healthcare system. Given the popularity of acupuncture, such difficulties have wide-ranging practical impact. In this section, we analyse some of these challenges and argue that they are partly due to the reducing of multiple nodes of tensions (social/professional/cultural/historical) into scientific and clinical forms of knowledge and evidence. In spite of such commonalities however, the framing of acupuncture as practice is also reflective of much broader questions of identities and boundary-making in relation to medical professions, healthcare systems, and their relation to the state (and state law), in France and the UK.

The contrasts between the French and UK systems are multiple, but two features stand out as far as regulation is concerned. First, the boundary between doctors and non-doctors, central to the French legal system, has not been particularly relevant in the UK as far as the social ordering of acupuncture is concerned. Secondly, the reliance on state law in France as a starting point for ordering healthcare practices differs strikingly from the more managerial approach that runs through the UK and NHS system – acupuncture is both affected by, and representative of, this latter divergence.

Such contrasts and the dilemmas raised by the regulation of acupuncture can be examined in relation to two core issues (themselves subdivided into several questions): first, is acupuncture sufficiently proven to be authorised, integrated into health systems, and publicly financed? Second, who should legitimately practice acupuncture? If France and the UK have adopted similar tools and (scientific) logics to address the first issue, their contrasting approach to health systems and their regulation has produced quite different responses to the second. In addition, both in France and in the UK responses to these two questions have, to date, continued to produce grey areas where markets, (il)legalities and professional self-regulation intersect.

### Science, tradition and regulation through knowledge(s)

As we sketched out in the ‘Introduction’ section, one of the particular features of acupuncture has been the duality of its routes to legitimacy: legitimacy for acupuncture is negotiated both through science and through tradition. Both in France and in the UK however, where health regulatory agencies have intervened to determine the place that acupuncture should occupy in the health system (both for funding and integration of practices), they have prioritised scientific modes of evaluation. In the UK, this has been through NICE guidelines and in France, most recently through INSERM reviews and evaluation. Such reliance on scientific evidence is to be expected: science is seen as providing the sort of objective tools that regulators should rely upon when making informed decisions. At the same time, law and STS scholarship have long demonstrated that the relationship between scientific knowledge and regulation is always more complex. Scientific evidence is, in itself, a social practice, and regulatory processes, rather than simply relying on it, shape what constitutes ‘valid science’. Rather than a process of information gathering, the relation between science and regulation is therefore better defined as one of ‘co-production’ and interrelation.^49^ Acupuncture is a useful site to observe such dilemmas. This is because, as others have demonstrated, it operates on a mode of knowledge-making that is not reducible to Western science or biomedicine, making visible some of the things we may otherwise take for granted about the universality of biomedicine’s modes of evidence.^50^

Even though regulators in France and the UK continue to see scientific evidence as key to determining the place of acupuncture in care, it is not entirely clear how scientific decision-making can be transferred to acupuncture: where the former assumes, for example, that bodies and minds can be approached separately, the latter’s attention on the circulation of energy calls for a very different logic.^51^ Secondly, where biomedicine operates from the assumption that bodily responses can be approached in a standardised or mechanical way, traditional Chinese medicine places a heavier emphasis on the individual nature of ill-being and of therapists.^52^ Thirdly, the possibility of testing the effect of needles implies the presence of a particular material object in the blinded trial, making ‘blindness’ materially difficult.^53^ As a result, tests such as double-blinded clinical trials, that assume a standardisation of treatment as the basis for evaluation, will not be able to replicate either the conditions of acupuncture practice, nor its traditional logic. All these elements contribute to destabilising the biomedical discourse around evidence and particularly the notion of placebo. The acupunctural interaction plays on a number of epistemological, social and material levels in which it is difficult to weight evidence against the comparative effects of a ‘placebo’– a concept that has also been critically challenged.^54^

As a result, even where scientific evidence is narrowed down and examined, the settlement that it may or may not provide for regulators leaves some ontological and practical issues unresolved. For example: the misalignment of its paradigms with those of acupuncture means that it may have a limited grip over practitioners and users; its approach to the placebo effect may mean that it does not sufficiently account for the relational element of treatment and fail to anticipate how those may be displaced onto other demands (and in turn other pressures on the health system and its costings); overall, it may fail to account for the complex institutional and socio-cultural arrangements that are at stake in the use of acupuncture. Indeed, even though France and the UK are relying on shared forms of scientific evidence for negotiating the place of acupuncture in the health system, evidence has yielded rather different outcomes in terms of the conditions of practice of acupuncture.

### Professional boundaries and the conditions of acupuncture

Aside from the question of scientific settlement and how it relates to the regulation of acupuncture, a core issue in France and the UK has been to determine what should be the conditions of its practice and professional framing. Here, France and the UK have most strikingly differed. The UK has opened the field of acupuncture to both healthcare professionals and other acupuncturists, and not placed doctors at the centre of practice. However, they do retain a significant, if normatively constrained, place as prescribers. France on the other hand has continued to hang onto the idea that only doctors should be able to provide medical treatment, and that acupuncture – in all its ambivalence and contested nature – should indeed constitute a ‘medical treatment’. This is reflective however of much broader contrast in medical regulation and professional dynamics in the two countries.

Fundamentally, the contrasts between the French and UK system for acupuncture are also visibly shaped by the particularities of their health systems, their histories, and the underlying relationships between state, professions and the law that they are built upon. The fragmented regulation of acupuncture in the UK, and its relative dislocation from doctors, is built against the particular form of managerial organisation of care that has come to define the NHS system. Centralised standards of care within the NHS and for its providers have constructed a particular form of biomedically framed acupuncture practice within the public system, while significant flexibility exists in the private sector. There, although professional standards and registration do exist, their uptake is voluntary, and their organisation separate to questions of knowledge and validity.^55^ Although this creates choice both for patients as to the type of acupuncture they may seek, and for practitioners in terms of their ability to practice in the way they prefer, it also leaves a number of questions open both in terms of equal access, and in terms of the guarantees that are offered to patients: effectively, the intervention of the state is mostly about framing the services they may purchase, as consumer, rather than engaging with the legitimate borders of care themselves.

Although the monopoly granted to doctors may signal a certain strength of the profession, and a certain independence, this is also partial, revealing an ambivalence in the relationship between doctors and the state that is deep-rooted in France.^56^ On the one hand, doctors have preserved both their exclusive right to diagnose and treat bodies, and their relative independence from direct clinical guidance from state institutions (in particular in private practice). At the same time, their everyday practice is also indirectly shaped by the conditions under which particular acts will be refunded by the social security system: the time that can be devoted to each consultation, the rates under which different acts can be refunded for patients, will all impact on what, in practice, doctors can sustain and offer. As increasing pressure is being placed by their peers on the value of their approach, doctor-acupuncturists will experience this remaining relationship to the state yet more strongly.

### Acupuncture, regulatory frictions and health system identities

In both France and the UK, negotiations around acupuncture have also been about the maintenance of boundaries. This plays out however in different ways. In the UK, approaches to acupuncture that do not meet the demands of the NHS (including for scientific proof) can be negotiated on the edge of publicly funded care through private providers.

In France, such ambivalence and gaps undoubtedly also exist, maybe even more strikingly than in the UK. Effectively, a whole section of acupuncture provision operates in a grey regulatory zone: illegal yet de facto tolerated; precarious yet widespread; non-legitimate (in the eyes of the state) and therefore not regulated. This last characteristic is at the core of the difficulty that public agents meet in approaching both CAM in general and acupuncture in particular. Any effort to regulate through the existing state channels is perceived as a form of implicit recognition of agents that the state is unwilling to ‘make space for’ in the health system. As only doctors can treat patients, envisaging professional regulation for others through state agencies is seen as risking the erosion of the fundamental principle of this medical monopoly. The status quo is therefore currently maintained of unrecognised and therefore unregulated practices, where professions are left to self-regulate ‘in the shadows of the state’, while remaining formally illegal.

In the UK, the assemblage of multiple forms of regulation has different side effects, since it allows a certain freedom for patients to choose their paths. However, it does so by segmenting the responsibilities that different actors take, often leaving the user in an uncertain position. The person can be considered as a patient, in the NHS public provision of care, but within the cost-efficiency logic of NICE; otherwise, she immediately becomes a consumer, since the provision of acupuncture becomes a commercial matter. As a consequence, not only the consumer/patient split destabilises her right to health and care, but it also exposes a crucial question that the recent regulatory debates on healthcare management in the UK have been addressing thoroughly, the ones around accountability, trust and transparency.

## Conclusions

Overall, the regulation of acupuncture in France and the UK remains an unsettled question. In the years following its increasing uptake by patients from the 1970s, acupuncture has been subject in both countries to new demands, new framings and new opportunities. At the same time, its folding within health systems has illustrated the frictions it creates in terms of regulatory knowledge and of professional practice. France and the UK have taken different paths to the organisation of acupuncture which seems to have generated curiosity and a degree of scepticism on both sides. The inherent logics of each system are indeed rooted deeply into different ways of doing ‘public healthcare’. However, commonalities in the challenges that remain are also strikingly visible. In both contexts, pockets of unregulated, or lightly regulated, provision continue to exist, where the state seems to have stopped interrogating the substantive question of how acupuncture in its diversity could be legitimately, and safely, practiced. A privileged attention to the question of proof and scientific evidence has rendered less visible the more difficult question of the social practice of acupuncture, and how best to organise it. If the question of whether acupuncture works ‘enough’ to be publicly funded has remained a clear item on policy agendas, the pragmatic question of how to regulate and organise it in its multiple forms of existence, even those that deviate from scientific evidence to embrace a more traditional understanding of the practice, is less carefully addressed in both national contexts.

## Notes

1. R. Bivins, ‘The Needle and the Lancet: Acupuncture in Britain, 1683–2000’, *Acupuncture in Medicine* 19(1) (2001), pp. 2–14; L. Candelise, *La médecine chinoise dans la pratique médicale en France et en Italie, de 1930 à nos jours. Représentations, réception, tentatives d’intégration* (PhD EHESS/Università degli Studi di Milano Bicocca, 2008).

2. L. Candelisa, *La médecine chinoise dans la pratique médicale en France et en Italie*; R. Guilloux, ‘L’acupuncture extrême-orientale face à la modernisation de la médecine occidentale (XVIIe-XIXe siècles)’, *Acupuncture et moxibustion* 6(4) (2008), pp. 294–300.

3. L.L. Barnes, ‘The Acupuncture Wars: The Professionalizing of American Acupuncture – A View from Massachusetts’, *Medical Anthropology* 22(3) (2003), pp. 261–301; F. Parent, ‘Seuls les médecins se piquent d’acupuncture?’, *Terrains & Travaux* 25(2) (2015), pp. 21–38.

4. B. Andrews. *The Making of Modern Chinese Medicine, 1850-1960*. (UBC Press, 2014); H. Chiang, ‘Historical Epistemology and the Making of Modern Chinese Medicine’ [Internet] (1 July 2015); Available at: https://www.manchesterhive.com/view/9781784991906/9781784991906.00009.xml (accessed 22 April 2019); E. Ernst, ‘The Recent History of Acupuncture’. *The American Journal of Medicine* 121(12) (2008), pp. 1027–1028; A. White and E. Ernst. ‘A Brief History of Acupuncture’. *Rheumatology* 43(5) (2004), pp. 662–663; Y. Zhuang et al. ‘Chapter One – History of Acupuncture Research’. *International Review of Neurobiology* 111 (2013), pp. 1–23.

5. L.L. Barnes, *Needles, Herbs, Gods, and Ghosts: China, Healing, and the West to 1848* (Harvard University Press, 2009).

6. J. Farquhar, ‘Knowledge in Translation: Global Science, Local Things’, *Medicine and the Politics of Knowledge* (2012), pp. 153–170; C. Kim and B. Lim, ‘Modernized Education of Traditional Medicine in Korea: Is It Contributing to the Same Type of Professionalization Seen in Western Medicine?’, *Social Science & Medicine* 58(10) (2004), pp. 1999–2008.

7. J. Almeida, P. Siegel and N. Filice De Barros, ‘Towards the Glocalisation of Complementary and Alternative Medicine: Homeopathy, Acupuncture and Traditional Chinese Medicine Practice and Regulation in Brazil and Portugal’, in C. Brosnan, P. Vuolanto and J.-A. Brodin Danell, eds., *Complementary and Alternative Medicine: Knowledge Production and Social Transformation* (Cham: Springer International Publishing, 2018), pp. 113–137; H.A. Baer et al., ‘The Drive for Professionalization in Acupuncture: A Preliminary View from the San Francisco Bay Area’, *Social Science & Medicine* 46(4) (1998), pp. 533–537; Barnes, ‘The Acupuncture Wars’; P.-H.D.E. Bruyn and E. Micollier, ‘The Institutional Transmission of Chinese Medicine: A Typology of the Main Issues’, *China Perspectives, Hong Kong: French Centre for Research on Contemporary China* 3 (2011), pp. 23–32; R. Frank and G. Stollberg, ‘Conceptualizing Hybridization: On the Diffusion of Asian Medical Knowledge to Germany’, *International Sociology* 19(1) (2004), pp. 71–88; N. Ijaz and H. Boon, ‘Safety as ‘Boundary Object’: The Case of Acupuncture and Chinese Medicine Regulation in Ontario, Canada’, in C. Brosnan, P. Vuolanto, and J.-A.B. Danell, eds. *Complementary and Alternative Medicine: Knowledge Production and Social Transformation* (Cham: Springer International Publishing, 2018), pp. 193–213; C.E. McClelland, *The German Experience of Professionalization: Modern Learned Professions and Their Organizations from the Early Nineteenth Century to the Hitler Era* (Cambridge University Press, 2002).

8. R. Frank and G. Stollberg, ‘Medical Acupuncture in Germany: Patterns of Consumerism among Physicians and Patients’, *Sociology of Health & Illness* 26(3) (2004), pp. 351–372.

9. J. Kim, ‘Beyond Paradigm: Making Transcultural Connections in a Scientific Translation of Acupuncture’, *Social Science & Medicine* 62(12) (2006), pp. 2960–2972; W.-J. Lin and J. Law, ‘A Correlative STS: Lessons from a Chinese Medical Practice’, *Social Studies of Science* 44(6) (2014), pp. 801–824; W.-J. Lin, ‘Shi (勢), STS, and Theory: Or What Can We Learn from Chinese Medicine?’, *Science, Technology, & Human Values* 42(3) (2017), pp. 405–428; K. Taylor, ‘Divergent Interests and Cultivated Misunderstandings: The Influence of the West on Modern Chinese Medicine’, *Social History of Medicine* 17(1) (2004), pp. 93–111.

10. S. Birch, T. Alraek and M. Soo Lee, ‘Challenges for Clinical Practice Guidelines in Traditional Medicines: The Example of Acupuncture’, *European Journal of Integrative Medicine* 8(4) (2016), pp. 332–336; R.W. Carrubba and J.Z. Bowers, ‘The Western World’s First Detailed Treatise on Acupuncture: Willem Ten Rhijne’s De Acupunctura’, *Journal of the History of Medicine and Allied Sciences* XXIX(4) (1974), pp. 371–398.

11. S. Tang, ‘‘From Outcast to Inboard’: The Transmission, Professionalisation and Integration of Acupuncture into British Medical Culture’, *Asian Medicine* 2(2) (e2018), p. 264.

12. A. Wahlberg, ‘Modernisation and Its Side Effects’ (PhD, The London School of Economics and Political Science (LSE), 2006), p. 48. Available at: http://etheses.lse.ac.uk/298/.

13. J.-S. Han and Y.-S. Ho, ‘Global Trends and Performances of Acupuncture Research’, *Neuroscience & Biobehavioral Reviews* 35(3) (2011), pp. 680–687; S. Nadimpalli, ‘Should the Use of Complementary and Alternative Medicine Be Restricted?’, *International Journal of Basic & Clinical Pharmacology* 5(5) (2017), pp. 1691–1694; S.-M. Wang, R.E. Harris, Y.-C. Lin and T.-J. Gan., ‘Acupuncture in 21st Century Anesthesia: Is There a Needle in the Haystack?’, *Anesthesia & Analgesia* 116(6) (2013), p. 1356; A. White and M. Cummings, ‘Inconsistent Placebo Effects in Nice – s Network Analysis’, *Acupuncture in Medicine* 30(4) (2012), pp. 364–365; P. White, ‘A Background to Acupuncture and Its Use in Chronic Painful Musculoskeletal Conditions’, *Journal of the Royal Society for the Promotion of Health* 126(5) (2006), pp. 219–227.

14. S.L. Cant and U. Sharma, ‘Professionalization of Complementary Medicine in the United Kingdom’, *Complementary Therapies in Medicine* 4(3) (1996), pp. 157–162; D.B. Clarke, M.A. Doel and J. Segrott, ‘No Alternative? The Regulation and Professionalization of Complementary and Alternative Medicine in the United Kingdom’, *Health & Place*, Special Section: Geographies of Health Knowledge/s, 10(4) (2004), pp. 329–338; N. Gale, ‘The Sociology of Traditional, Complementary and Alternative Medicine’, *Sociology Compass* 8(6) (2014), pp. 805–822; A. Givati, ‘Performing ‘Pragmatic Holism’: Professionalisation and the Holistic Discourse of Non-Medically Qualified Acupuncturists and Homeopaths in the United Kingdom’, *Health* 19(1) (2015), pp. 34–50; A. Givati and K. Hatton, ‘Traditional Acupuncturists and Higher Education in Britain: The Dual, Paradoxical Impact of Biomedical Alignment on the Holistic View’, *Social Science & Medicine* 131 (2015), pp. 173–180.

15. M. Wiese, C. Oster and J. Pincombe, ‘Understanding the Emerging Relationship between Complementary Medicine and Mainstream Health Care: A Review of the Literature’, *Health* 14(3) (2010), pp. 326–342.

16. J. Adams, G. Easthope and D. Sibbritt, ‘Exploring the Relationship Between Women’s Health and the Use of Complementary and Alternative Medicine’, *Complementary Therapies in Medicine* 11(3) (2003), pp. 156–158; N. Faass, *Integrating Complementary Medicine into Health Systems* (Jones & Bartlett Learning, 2001); D. Hollenberg, ‘Uncharted Ground: Patterns of Professional Interaction among Complementary/Alternative and Biomedical Practitioners in Integrative Health Care Settings’, *Social Science & Medicine* 62(3) (2006), pp. 731–744; T.J. Kaptchuk and F.G. Miller ‘What Is the Best and Most Ethical Model for the Relationship Between Mainstream and Alternative Medicine: Opposition, Integration, or Pluralism?’, *Academic Medicine* 80(3) (2005), pp. 286–290.

17. E. Cloatre, ‘Law and ANT (and Its Kin): Possibilities, Challenges and Ways Forward’, *Journal of Law and Society* 45(4) (2018), pp. 646–663.

18. S. Jasanoff, ‘Ordering Knowledge, Ordering Society’, in *States of Knowledge: The Co-Production of Science and Social Order* (Routledge, 2006).

19. R. Bivins, *Alternative Medicine?: A History* (Oxford University Press, 2010).

20. S. Raman, ‘Science, Uncertainty, and the Normative Question of Epistemic Governance in Policymaking’, in *Knowledge, Technology and Law* (Routledge, 2014).

21. Ethics approval granted by the University of Kent on the 1st March 2017.

22. Wellcome Investigator Award 200380/Z/15/Z, ‘Law, Knowledges and the Making of “Modern” Healthcare’ (2017–2022).

23. R.E. Bivins, ‘Acupuncture and Innovation: “New Age” Medicine in the NHS’, in R.W. Carrubba, J.Z. Bowers and J. Stanton, eds. *Innovations in Health and Medicine: Diffusion and Resistance in the Twentieth Century* (New York, NY: Routledge, 2002): pp. 84–105. Available at: http://orca.cf.ac.uk/3887/; R. Carrubba and J.Z. Bowers, ‘The Western World’s First Detailed Treatise on Acupuncture’; J. Farquhar, *Knowing Practice: The Clinical Encounter of Chinese Medicine*, 1994.

24. This was the case, for example, in the Gateway Clinic in Lambeth, London, and the Complementary Therapy and Research Unit in Lewisham as sites of experimentation in the primary healthcare structures of the NHS.

25. J. Alltree, ‘Physiotherapy and Acupuncture: Practice in the UK’, *Complementary Therapies in Medicine* 1(1) (1993), pp. 34–41; R. Bivins, ‘Imagining Acupuncture: Images and the Early Westernization of Asian Medical Expertise’, *Asian Medicine* 7(2) (2012), pp. 298–318.

26. See House of Lords, *Science and Technology* – *Sixth Report,* 2000. Available at: https://publications.parliament.uk/pa/ld199900/ldselect/ldsctech/123/12301.htm.

27. See Great Britain and Herbal Medicine Steering Group on the Statutory Regulation of Practitioners of Acupuncture Traditional Chinese Medicine and Other Traditional Medicine Systems Practised in the UK, *Report to Ministers from the Department of Health Steering Group on the Statutory Regulation of Practitioners of Acupuncture, Herbal Medicine, Traditional Chinese Medicine and Other Traditional Medicine Systems Practised in the UK.* (Aberdeen: Robert Gordon University, 2008).

28. See Great Britain and Steering Group on the Statutory Regulation of Practitioners of Acupuncture, *Report to Ministers from the Department of Health Steering Group*.

29. F.L. Bishop et al., ‘Patients as Healthcare Consumers in the Public and Private Sectors: A Qualitative Study of Acupuncture in the UK’, *BMC Health Services Research* 11(1) (2011), p. 129; H. MacPherson, N. Sinclair-Lian and K. Thomas, ‘Patients Seeking Care from Acupuncture Practitioners in the UK: A National Survey’, *Complementary Therapies in Medicine* 14(1) (2006), pp. 20–30.

30. British Medical Association, *Acupuncture: Efficacy, Safety and Practice* (Routledge, 2005); P. Baldry, ‘The Integration of Acupuncture within Medicine in the UK – The British Medical Acupuncture Society’s 25th Anniversary’, *Acupuncture in Medicine* 23(1) (2005), pp. 2–12; White, ‘A Background to Acupuncture’.

31. Department of Health, *Enabling Excellence: Autonomy and Accountability for Health and Social Care Staff* (2011). Available at: https://www.gov.uk/government/publications/enabling-excellence-autonomy-and-accountability-for-health-and-social-care-staff.

32. Birch, Alraek and Lee, ‘Challenges for Clinical Practice Guidelines’; J. Dale, ‘Acupuncture Practice in the UK. Part 1: Report of a Survey’, *Complementary Therapies in Medicine* 5(4) (1997), pp. 215–220; K.J. Hunt et al., ‘Complementary and Alternative Medicine Use in England: Results from a National Survey’, *International Journal of Clinical Practice* 64(11) (2010), pp. 1496–1502.

33. F.L. Bishop, S. Zaman and G.T. Lewith, ‘Acupuncture for Low Back Pain: A Survey of Clinical Practice in the UK’, *Complementary Therapies in Medicine* 19(3) (2011), pp. 144–148; J. Inman and O.P. Thomson, ‘Complementing or Conflicting? A Qualitative Study of Osteopaths’ Perceptions of NICE Low Back Pain and Sciatica Guidelines in the UK’, *International Journal of Osteopathic Medicine* 31 (2019), pp. 7–14; L. Lai, ‘NICE Should Reconsider Its Recommendation to Withdraw Acupuncture from Its 2016 Guidelines on Low Back Pain and Sciatica’, *European Journal of Integrative Medicine* 8(4) (2016), pp. 329–331; NICE, ‘Overview/Low Back Pain and Sciatica in over 16s: Assessment and Management/Guidance/NICE’ (2016). Available at: https://www.nice.org.uk/guidance/ng59.

34. J. Dale, ‘Acupuncture Practice in the UK. Part 2: Making Sense of Diversity’, *Complementary Therapies in Medicine* 5(4) (1997), pp. 221–225; MacPherson, Sinclair-Lian, and Thomas, ‘Patients Seeking Care from Acupuncture Practitioners’.

35. L. Candelise, La médecine chinoise dans la pratique médicale en France et en Italie; *Bulletin Amades* 81 (2010). Available at: http://amades.revues.org/1139; R. Guilloux, ‘Évolution de la “tradition” dans la réception de l’acupuncture chinoise en France (1860-1980)’, *Revue d’Anthropologie des Connaissances* 5(1) (2011), pp. 13–40.

36. M. Ramsey, ‘Alternative Medicine in Modern France’, *Medical History* 43(3) (1999), pp. 286–322.

37. E. Cloatre, ‘Regulating Alternative Healing in France and the Problem of ‘Non-Medicine’, *Medical Law Review* 27(2) (2019) pp. 189–214.

38. Code de la Santé publique Art.L4161-1 to L4161-6.

39. Cloatre, ‘Regulating Alternative Healing in France’; Parent, ‘Seuls les médecins se piquent d’acupuncture’; C. Barry, V. Seegers, J. Gueguen, C. Hassler, A. Ali and B. Falissard ‘Evaluation de l’efficacité et de la sécurité de l’acupuncure’, *Rapport INSERM* (2014). Available at: https://www.inserm.fr/sites/default/files/2017-11/Inserm_RapportThematique_EvaluationEfficaciteSecuriteAcupuncture_2014.pdf.

40. Décret n° 2008-863 du 27 août 2008.

41. Parent, ‘Seuls les médecins se piquent d’acupuncture’.

42. For an early summary of these rationales, see, for example, Ministère des Affaires Sociales et de la Solidarité nationale (1986).

43. Candelise, *La médecine chinoise dans la pratique médicale en France et en Italie*.

44. Op. cit.

45. Tribune, 18th March 2018, ‘L’appel de 124 Professionnels de la Santé cone les “Médecines Alternatives”’. Available at: http://www.lefigaro.fr/vox/societe/2018/03/18/31003-20180318ARTFIG00183-l-appel-de-124-professionnels-de-la-sante-contre-les-medecines-alternatives.php.

46. Cloatre, ‘Regulating Alternative Healing in France’.

47. Parent, ‘Seuls les médecins se piquent d’acupuncture’; Cloatre, ‘Regulating Alternative Healing in France’.

48. Barry et al., ‘Evaluation de l’efficacité et de la sécurité de l’acupuncture’.

49. Jasanoff, ‘Ordering Knowledge, Ordering Society’.

50. Barnes, *Needles, Herbs, Gods, and Ghosts;* Farquhar and Intrepid, *Knowing Practice*.

51. Bruyn, ‘The Institutional Transmission of Chinese Medicine’; Farquhar and Intrepid, *Knowing Practice*.

52. Barnes, *Needles, Herbs, Gods, and Ghosts*; J. Fadlon, ‘Meridians, Chakras and Psycho-Neuro-Immunology: The Dematerializing Body and the Domestication of Alternative Medicine’, *Body & Society* 10(4) (2004), pp. 69–86.

53. White and Cummings, ‘Inconsistent Placebo Effects in Nice’.

54. C.A. Barry, ‘The Role of Evidence in Alternative Medicine: Contrasting Biomedical and Anthropological Approaches’, *Social Science & Medicine* 62(11) (2006), pp. 2646–2657; P. Friesen, ‘Mesmer, the Placebo Effect, and the Efficacy Paradox: Lessons for Evidence Based Medicine and Complementary and Alternative Medicine’, *Critical Public Health* 29(4) (2019), pp. 435–447.

55. Bishop et al., ‘Patients as Healthcare Consumers in the Public and Private Sectors’.

56. M. Steffen, ‘The French Health Care System: Liberal Universalism’, *Journal of Health Politics, Policy and Law* 35(3) (2010), pp. 353–387.

